# A teledentistry care model for older populations in remote settings in Chile using satellite communication technology

**DOI:** 10.3389/froh.2025.1699401

**Published:** 2025-12-04

**Authors:** Víctor Beltrán, Leonardo López, Pablo Acuña-Mardones, Roberto Silva, Claudia Acevedo, Jaime Bustos, Claudio Alarcón, Randal von Marttens, Iris Espinoza, Rodrigo Mariño

**Affiliations:** 1 Universidad de La Frontera, Centro de Investigación e Innovación en Odontología Clínica (CIDIC), Facultad de Odontología, Temuco, Chile; 2 Universidad de La Frontera, Centro de Excelencia en Medicina Traslacional (CEMT), Temuco, Chile; 3 Universidad de La Frontera, Núcleo Científico y Tecnológico en Biorecursos (BIOREN), Temuco, Chile; 4 Universidad de La Frontera, Centro de Excelencia en Física e Ingeniería en Salud (CFIS), Facultad de Ingeniería y Ciencias, Temuco, Chile; 5 Universidad de La Frontera, Instituto de Informática Educativa, Temuco, Chile; 6 Universidad de La Frontera, Doctorado en Ciencias Morfológicas, Facultad de Medicina, Temuco, Chile; 7 Fuerza Aérea de Chile, Santiago, Chile; 8 Universidad de La Frontera, Departamento de Ingeniería Industrial y de Sistemas, Facultad de Ingeniería y Ciencias, Temuco, Chile; 9 Universidad de La Frontera, Programa de Magíster en Odontología, Facultad de Odontología, Temuco, Chile; 10 Departamento de Patología y Medicina Oral, Facultad de Odontología, Universidad de Chile, Santiago, Chile; 11 Monash Health, Monash Health Dental Services, Dandenong, VIC, Australia; 12 Melbourne Dental School, The University of Melbourne, Parkville, VIC, Australia; 13 Department of Conservative Dentistry and Oral Health, Riga Stradiņš University, Riga, Latvia

**Keywords:** telehealth, teledentistry, oral health, satellite connection, rural and remote

## Abstract

**Objectives:**

The aim of this study is to provide initial evidence of the impact of teledentistry, using satellite communication technology to improve access to oral healthcare in underserved populations in rural and remote areas.

**Methods:**

A pilot model of teledentistry care for older adults, incorporating technological and clinical components with a satellite solution for areas without connectivity, was evaluated in the Los Lagos Region, Chile. This was classified in terms of the sequential industrial modernization framework and technology types.

**Results:**

Thirty-one older adults participated in this evaluation, with a mean age of 66.3 years. The majority (56%) had not visited a dentist in over five years. The average DMFT index was 24.2 teeth, with almost half of the participants (45.4%) presenting unmet restorative needs, and 83.3% required prosthetic appliances. A large proportion of the sample requiring a referral for emergency treatment. Most consultations (84%) were for emergency care, predominantly due to dental pain. The strategy was classified as part of the Industry 4.0 trend of modernization, with elements of the Industry 3.0.

**Conclusion:**

This piloted model of care can enhance care access and improve health outcomes for isolated populations. This innovative model of care ensured that all participants received oral health assessments, addressing the need for the systematic identification of older adults at risk of oral health emergencies. Future directions could include the integration of contextual artificial intelligence and expansion into other underserved areas, reinforcing the role of digital ecosystems in enhancing access to care and reducing health disparities.

## 1 Introduction

Around the world, significant disparities in health outcomes between urban and rural populations persist, particularly in relation to oral healthcare (1–4). Access to oral healthcare in rural areas is often constrained by geographical and transportation barriers, local shortages of oral health professionals, and inadequate healthcare infrastructure (5–8). Addressing these challenges, along with broader socioeconomic inequities and social determinants of health, is essential to improving access and reducing inequalities in oral health services. In particular, the geographic isolation of rural communities presents unique and persistent obstacles to equitable oral healthcare delivery.

In recent years, the landscape of healthcare delivery has undergone profound transformation. The rise of digital health and telehealth platforms offers several advantages for health promotion and serves as an effective point of entry for patients into the public oral healthcare system (9, 10). This also facilitates the identification of at-risk individuals, overcoming traditional geographic and logistical barriers (11–13), and provides valuable opportunities for teaching and research. This approach was previously implemented in two studies that applied teledentistry models for older adults living in remote areas of Chile. The first implemented an asynchronous (store-and-forward) teledentistry network during the COVID-19 pandemic (14), in which general dentists collected clinical data and digital images in mobile units and referred cases remotely to specialists. This model effectively maintained access to oral care for older adults with urgent or priority needs. The subsequent study extended the model to rural Mapuche communities (15), reporting a high burden of oral disease and demonstrating the usefulness of remote specialist input in reducing travel barriers.

However, despite the potential of telehealth to bridge gaps in access to oral healthcare, many rural and remote communities continue to face significant challenges that hinder the effective implementation of these solutions. A major barrier to the successful deployment of telemedicine in such regions is the lack of reliable internet connectivity. Rural and remote areas are often characterized by sparse populations and vast distances, which can make it economically unfeasible for investment in the necessary infrastructure to provide internet services. As a result, residents in these communities are left without access to essential telehealth services, thereby exacerbating existing health disparities. Furthermore, the limitations in connectivity not only affect telehealth but also hinder the overall socioeconomic development of these areas, as access to information and communication technologies (ICT) is increasingly recognized as a critical determinant of health and well-being (16).

Satellite communication offers a practical solution to providing internet access where traditional networks fail. The integration of satellite technology in teledentistry can serve as an essential element of reducing the isolation experienced in rural areas. This study aims to generate preliminary evidence for using satellite communication technology to support the expansion of digital oral health models, improving access to care for rural and remote populations in Chile, particularly for older adults with limited internet connectivity. Additionally, to highlight the digital transformation and its relation to industrial revolutions, the study will examine the technologies developed for the system within the framework of Industry 4.0 and the Sequential Industrial Modernisation Framework to evaluate its potential contribution to the professional practice of dentistry.

## 2 Materials and methods

This section is organized into three parts. The first details a teledentistry care model developed for older adults in remote areas, technological architecture and security and the satellite solution aimed at providing connectivity. The second part outlines the clinical assessment methods. The last part describes the classification in terms of the Industry 4.0, the sequential industrial modernization framework and technology types, used to analyse how innovative digital oral health applications can improve access to care using.

### 2.1 Part 1 integrated teledentistry ecosystem for older adults

The teledentistry care model developed for older adults integrates technological, organisational, and clinical components to address the specific challenges faced by this vulnerable population, particularly in remote areas with limited access to oral health care. The structure of the model is based on a protocolised sequence of steps, starting with the identification and recruitment of older adult patients. Recruitment of participants was conducted in collaboration with institutional and community organizations representative of the rural population in Chile. Primarily, the National Service for Older Adults (SENAMA: In Spanish: Servicio Nacional del Adulto Mayor). SENAMA's regional database provides up-to-date, comprehensive information about older adults. Identification of potential participants was based on the inclusion criteria applied to these records and data from local community organizations that periodically assess health needs, including oral health needs. These organizations generate lists for priority healthcare, which were supplemented by interviews with local older adults' associations, community leaders, and local teams, confirming eligibility and willingness to participate. Thus, recruitment was systematic, with institutional support and active community participation, ensuring a representative and contextualized sample.

Patient admission included collecting sociodemographic and medical information via remote or in-person interviews. This stage was limited to registration and scheduling of visits. Clinical care was provided in mobile units, community centers, or in the patient's homes, tailored to patients' mobility levels. Dentists performed examinations using digital tools, enabling synchronous or asynchronous referrals to specialists (e.g., oral pathologist, periodontologist, radiologist, geriatrics, etc.).

The Teleplatform for Geriatric and Dental Specialties (In Spanish: Teleplataforma de Especialidades Geriátricas y Odontológicas (TEGO) formed the core infrastructure of the model, facilitating the management of clinical information, teleconsultations, and logistical aspects such as appointment scheduling and monitoring patient follow-ups, enhancing decision-making and continuity of care for older adults. The model prioritises connectivity within the care network by allowing general dentists to share information with specialists and remote support teams. Through virtual meetings, screen sharing, and secure messaging, it facilitated case registration, follow-up, and the export of clinical reports for effective oral health management.

### 2.2 Operational flow and structure of the TEGO platform

The TEGO platform operated as a web-based system that facilitates the management and implementation of dental care for older adults through digital modules that allow the storage of anamnesis records for patients treated in mobile units (14). The operational process begun with the enrolment of older adults in the TEGO platform by trained staff, who registered and scheduled mobile care appointments in their areas of residence. The platform recognises user profiles with specific functionalities according to their responsibilities.

During the face-to-face consultations, a digital anamnesis was prepared, which was organised in a clinical file composed of a general anamnesis, medical-geriatric anamnesis, and dental-geriatric anamnesis. The dental-geriatric anamnesis was supported by a virtual 3D phantom, allowing the integration of observations, images, or photographic records at specific anatomical sites, simulating the standardised orofacial structure of an adult. This record facilitated communication between the general dentist and specialists from various dental and medical-geriatric fields.

The workflow concluded in the clinical records module, where all generated information converged and allowed interaction between the general dental practitioner and the assigned specialists, who may supplement the information with medical examinations and monitor the patient's progress. The TEGO platform has an associated mobile app focused on oral health education interventions for older adults.

### 2.3 Technological architecture and security

The TEGO platform utilized a classical three-layer web architecture, ensuring a secure and scalable technological environment for managing clinical information. The Presentation Layer *(FrontEnd)*, developed with React.js, featured user-friendly interface and integrates 3D models, enhancing visualization and communication between dentists and specialists through interactive representations of anatomical structures. The Business Logic Layer, built on Node.js, managed application logic, data interchange, and security, using Express for efficient communication, MySQL for secure database interaction, bcrypt for password encryption, and jsonwebtoken for access control. Only authorised general dentists and specialists can view or modify patient records. Authentication is role-based and managed through encrypted credentials using bcrypt and jsonwebtoken protocols; no external or public users can access clinical data.

The Data Layer stored clinical and administrative information in a MariaDB database on a cloud-based server, ensuring data confidentiality and integrity with robust security measures, including encryption and regular updates. Additionally, the TEGO mobile application, created with Flutter, supports oral health education and preventative interventions, allowing patients access to appointment information and educational resources while promoting adherence to treatment (17). Overall, the platform emphasizes security and compliance with health information protection regulations.

The TEGO platform was designed for modular expansion and scalability, facilitating the addition of new clinical modules and integration with external health information systems, such as national health registries. Its technological stack ensures that future needs, including increased user capacity and artificial intelligence algorithms for clinical decision support, could be accommodated without affecting performance or security.

### 2.4 Satellite connectivity for remote areas

To ensure access in areas without terrestrial connectivity, a portable satellite ground station was available to support real-time tele-dentistry. This system included a 1.8 m Ku-band parabolic antenna, Block Up Converter (BUC), Low Noise Block downconverter (LNB), SCPC modem, Cisco switch, two IP phones, coaxial/RF cabling, and tactical transport cases. The system operated on a 15 MHz channel within a 36 MHz transponder, delivering an effective throughput of approximately 10 Mbps and a latency of 550 ms (See Table 1).

**Table 1:** Performance metrics of the satellite system.

Parameter	Measurement
Data throughput	∼10 Mbps (15 MHz carrier)
Latency	∼550 ms (RTT)
Coverage	Latin America
Useful life	≥15 years

Communications were provided via the geostationary satellite Eutelsat 117 West B, positioned at 117° W and operating in the Ku band. Launched in 2016 on the Boeing BSS 702SP platform, with a useful life of over 15 years, it features 48 transponders and coverage across Mexico, Central America, the Caribbean, the Andean region, and the Southern Cone. The system provides robust, reliable, and continuous coverage for the operation of TEGO in remote locations. The Eutelsat 117 West B satellite provides complete coverage of continental Chile, including the municipality of Cochamó where data were collected; therefore, the entire study area was within the operational footprint of the portable ground station.

### 2.5 Part 2 clinical assessments

This assessment employed a cross-sectional design. With the ethics' approval granted by the Ethics Committee of the Universidad de La Frontera (UFRO) (ID: 169/23, approved on 18 March 2024), residents of the municipality of Cochamó in the Los Lagos Region of Chile were invited to participate in this assessment. This location was selected due to the area's limited access to the internet and for reasons of convenience and the working relationship established with the municipality. Data was collected between September and October 2024.

The inclusion criteria consisted of individuals aged 60 years or older, able to provide informed consent and follow instructions. Participants were dentate, defined as having one or more natural teeth. By focusing on dentate participants, the study aimed to explore how environmental factors, such as access to oral healthcare, and individual contextual factors, like socioeconomic status, influenced current oral health status. Informed consent was obtained from all participants prior to their inclusion in the study.

Oral examinations were carried out by one trained and calibrated dental examiner (RvM). The examiner received training and an online calibration exercise involving the review of 20 cases. The Intra-examiner reliability, measured using Cohen's Kappa statistics, exceeded 0.90, indicating an almost perfect level of agreement, as per Landis and Koch criteria (18).

Oral examinations were conducted in an approved by the regional Health Authority mobile dental clinic. Standardization of conditions during evaluations and treatments was ensured, including appropriate lighting, infection control protocols were followed in accordance with current health regulations to provide a safe environment for the evaluations.

Data collection comprised assessments of the following domains:

1. Sociodemographic characteristics: Participants’ age, sex, and educational level were recorded, categorized as “No formal education or complete primary education or less,” “Incomplete or complete secondary education,” and “Post-secondary education.”
2. Use of Oral Healthcare Services: Dental service utilization was assessed by participants indicating the time since their last dental visit, with response options: “12 months or less”, “12 months to 2 years”, “2 to 4 years”, “more than 4 years.” For this analysis, dental attendance was categorized as having visited a dentist within the last 12 months (Yes/No).
3. Clinical Data and Oral Health Status: Clinical data included:
  a. Dental status: the number of decayed teeth (DT), filled teeth (FT), and the number of natural teeth present were assessed according to the World Health Organization criteria for Oral Health Surveys (19). To further explore dental health status, the dental caries assessment included the proportion of unmet restorative needs. A restorative unmet normative needs index was calculated by dividing the number of decayed surfaces by the total number of decayed and filled surfaces: [DS/(DS + FS)] (20).
  b. Prosthetic Status: Evaluated normative need for dental prostheses, categorized as “Not needed or in good condition,” “Needs repairs,” “Needs one” or “Replacement needed.”
  c. Periodontal status was assessed using three indicators: 0 = sound; 1 = visual presence of gingivitis; 2 visual and tactile presence of periodontitis, using the CPI Probe (19).
  d. Urgency of Treatment: Recorded in four urgency levels: 1 = No urgency; 2 = Low urgency; 3 = Advanced urgency; and 4 = High urgency (19).

During clinical examinations, patients experiencing pain were treated without delays to in order to relieve their discomfort, including palliative care when needed. Cases requiring further treatment were referred to the oral health specialty clinic at the University of La Frontera, to ensure timely and appropriate follow-up. Moreover, participants had access to treatment and prosthetic rehabilitation at no cost to them. These services were provided under the comprehensive care program, ensuring that all diagnosed patients received necessary attention, including prosthetics, through institutional and public resources.

The costs of these referrals were either waived or covered by the project. Treatments were provided at the UFRO dental specialties clinics, thus ensuring timely and appropriate follow-ups.

The Statistical Analysis presents basic descriptive statistics for selected sociodemographic factors, dental status, unmet restorative and prosthetics needs, and periodontal status. Descriptive statistics included sociodemographic and oral health data, including means, standard deviations, frequency distributions. All statistical analyses were performed using SPSS Statistics (Version 30.0).

### 2.6 Part 3 classification in terms of the industry 4.0, the sequential industrial modernization framework and technology types

Industry 4.0, initiated in Germany in 2011 as a governmental program for industrial modernization, represents the integration of emerging technologies across various sectors, including, healthcare (21), education (22), and the pharmaceuticals (23, 24), emphasizing the digital transformation of traditional professional practices. The concept has led to terms like Industry 3.0 and Industry 5.0, associated with the Third and Fifth Industrial Revolutions. These terms have been used to classify developments and practices in the Sequential Industrial Modernisation Framework (25–27).

The classification in the study categorized technologies into five types based on their inputs and outputs: T1.0 (non-electrical), T2.0 (analogue electric), T3.0 (digital data), T4.0 (digital information), and T5.0 (digital contextual intelligence). Each technology was analysed according to its characteristics and classified across different industry levels. For example, “electronics” applies to both Industry 2.0 and Industry 3.0. The system's classification is determined by scoring, identifying its association with one or more Industry levels and Technology Types (25–27).

In this work the developed platform was analysed in terms of the Industry 4.0 concept in the Sequential Industrial Modernization Framework, and by a system-theory-based approach that considers the characteristics of its inputs and outputs (25–27).

## 3 Results

### 3.1 Clinical assessments

A total of 31 older adults agreed to participate in the study. Of these, 7 were excluded due to incomplete data. The remaining 24 participants were clinically examined and interviewed. Mean age was 66.3 (SD 4.7) years, with 61.4% women. According to the time interval since the last dental visit: 28% reported having been to the dentist in the previous 12 months. Another 16.0% reported their last visit to the dentist being more than 12 months and less than two years ago. More importantly, more than half of the sample (56.0%) reported not having been to the dentist within the last five years (20.0%), or longer (36.0%).

The mean number of teeth with dental caries history (i.e., DMFT index) was 24.2 (SD 4.0) teeth. Unmet restorative needs were high. A significant portion of the participants (45.4%) reported unmet restorative needs. Additionally, the majority of participants (83.3%) required a prosthetic appliance; of these, 85% indicated needs for repairs, replacements, or that one to be made, highlighting a continuous cycle of unmet needs that could lead to further complications in oral health.

Regarding the periodontal status, two of participants were found to have no periodontal needs (8.3%). Gingivitis was observed in 16 participants (66.7%%), and six (25.0%) of the sample had periodontitis.

**Figure 1:**
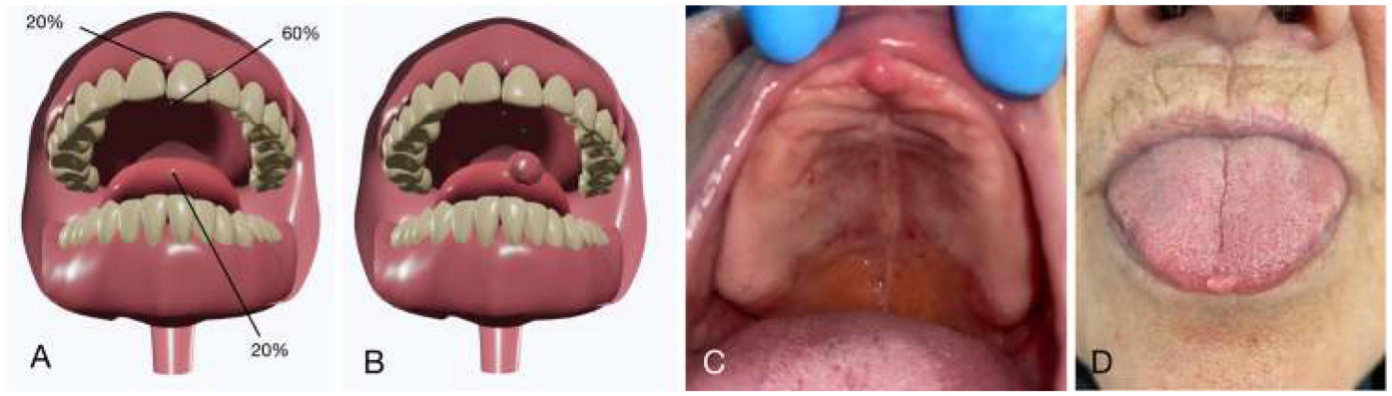
Localisation of intraoral lesions identified during examination. **(A,B)** 3D models showing lesion sites and frequencies. **(C,D)** Clinical records illustrating lesions at each location.

Data show that a large majority (84%) of consultations were for emergency care, with two-thirds of these emergencies due to dental pain. Consequently, by level of emergency, the majority had some level of emergency, although the majority was low level (83.9). Additionally, 9.7% of the participants showed no emergency. Three participants, (9.7%) were able to receive an online referral and consultation with an oral pathologist for potentially life-threatening conditions. All conditions were documented and indexed in a standardized 3D model of oral soft tissues and mucosa (Figure 1).

### 3.2 Satellite connection

As previously stated, the direct outcome concerning satellite coverage within the study area demonstrated enhanced access to oral healthcare services reliant on this technology. In Chile, the area encompassed by the satellite signal corresponds to 100% of the continental territory, including the Cochamó municipality. While there are some attenuation effects towards the extreme southern regions, these phenomena do not occur at the latitude of our area of interest.

Furthermore, there were no reported service failures during the period of data collection under analysis. Although some reports of intense solar events were recorded, resulting in G3-level geomagnetic storms, which may induce temporary disturbances in communications and minor disruptions in links, no critical failures were reported for the Eutelsat 117° West satellite. The system's resilience and redundancy allowed normal operations during data collection.

### 3.3 Classification in terms of the industry 4.0 concept

The technologies that are part of the developed system were identified and then classified (Table 2). The results show that most of the identified elements are part of Industry 4.0 (7/17), followed by Industry 3.0 (3/10). This allows classification of the presented system as part of the Industry 4.0 trend of modernization, with elements of the Industry 3.0.

**Table 2:** Identification of technologies and industry level associated.

Technology identified	Technologies identified/total per level	Industry level
–	0/2	Industry 1.0
Electricity, Electronics	2/6	Industry 2.0
Electronics, information technologies, communication technologies	3/10	Industry 3.0
Internet-based services, cloud computing, simulation, cybersecurity, mobile devices and applications	7/17	Industry 4.0
–	0/4	Industry 5.0

The input-output signals of the platform were identified from the point of view of the professional dentist interacting with the system. From these inputs and outputs, only digital technology was considered, as the platform requires interaction through the web-based platform. From this perspective, the system could also be considered as part of the Industry 4.0 levels.

## 4 Discussion

The clinical data presented several noteworthy insights regarding the oral health needs of participants. They suggest variability in the levels of access to oral healthcare, for example, only 28% had visited a dentist in the last 12 months, which is considered an optimal dental visits frequency (28). More alarmingly, 56.0% had not visited a dentist in at least five years. Nonetheless, with the implementation of this model of care, even those with less-than-optimal dental visits frequency received a dental examination and an assessment of their oral health needs.

A critical unmet need was the lack of systematic identification for older adults “at risk” of dental emergencies due to dental pain. The findings highlight persistent inequities in access to dental care. Although these emergencies may not be life-threatening, they can greatly affect an individual's quality of life, nutrition, and overall well-being. The emergency data does not exclude those with more advanced periodontal disease (25%), whose conditions could trigger acute episodes, suggesting many seek care reactively, potentially worsening their dental health over time. In addition to emergency referrals, a large portion of participants required referrals to general practitioners for preventive, restorative, periodontal, oral surgery, and prosthodontic care.

Treating dental emergencies poses significant costs for both individuals and society, particularly affecting older adults in rural and remote areas who are less likely to receive regular oral examinations. This leads to missed opportunities for early identification and for addressing their needs under conventional care models, even where teledentistry is available. It was only when the model was enhanced to include the alternative of satellite connectivity that these challenges were overcame, enhancing equitable access to oral healthcare services in underserved regions. This integration ensures that patients receive necessary care through real-time consultations, remote diagnoses, and ongoing monitoring, effectively meeting the oral health needs of those with limited access to traditional healthcare services.

Although Chile leads Latin America in internet connectivity (29), a significant proportion of the population, especially in rural and remote areas, remains uncovered, mainly due to the lack of broadband infrastructure rather than cost alone. According to national reports, in some rural regions, broadband coverage does not exceed 50%, and the challenge of deploying infrastructure in isolated areas is a major barrier to universal connectivity (30).

The present initiative was successfully applied in a study aimed at evaluating teledentistry for older adults residing in remote regions of Chile. The initiative builds on previous experiences (31) by establishing a model in which the initial contact with the healthcare system occurs in areas with limited or no connectivity. The study also included the provision of oral health care information and emergency dental services for those requiring immediate attention.

Developing or enhancing digital models could benefit those living in rural areas, with no permanent access to oral health services. Additionally, it is envisaged that, based on the results of this pilot model, dental public health strategies and oral health professional training programs could be developed. Accordingly, this study constitutes an initial yet significant contribution to research into oral health promotion. It complements existing data on oral health among older adults and offers implications for policy, research, and practice regarding the promotion of oral health and the delivery of care for this population. Furthermore, it is anticipated that the results of this pilot model will inform the development of public dental health strategies and training programmes for oral health professionals.

The present study also highlights the potential of targeted and tailored interventions for the reduction of discrimination and inequalities under the *Leave No One Behind* principle, a central component of the 2030 Sustainable Development and its Sustainable Development Goals (SDGs) agenda (32, 33). In fact, digital health is related to several United Nations SDG goals, including education (Goal 4), gender equality (Goal 5), infrastructure (Goal 9—ensuring universal and affordable internet access), and partnerships for implementation and diffusion of information and communications technologies and global interconnectedness (Goal 17) (34).

In the context of Industry 4.0, this work contributes to the emerging concept of Dentistry 4.0 (35, 36) by integrating advanced technologies into its design. The system incorporates key components such as internet-based services accessible via satellite connectivity, cloud computing for remote data management, and cybersecurity embedded within its framework. Additionally, simulation functionalities are included through 3D modelling. While the system represents significant progress towards cyber-physical systems—where physical and digital domains merge—the full integration of these domains is not yet complete. The study aligns with the Sequential Industrial Modernization Framework, highlighting digital information as a critical input-output signal in dental practice. Although the findings demonstrate the potential for digital modernization in dentistry, they also indicate the need for further technological integration and the incorporation of contextual artificial intelligence to achieve the Industry 5.0 level, outlining opportunities for future research and development in the field.

While this study offers valuable insights into the technology proficiency, and potential adoption using these technologies in populations with no or unreliable connectivity, it is not without its limitations. Firstly, although valuable insights have been gained about the oral health of this population, it must be noted that the purpose was not to obtain a definitive oral health profile of the older adult population living in remote geographic areas of Los Lagos Region. The findings offer a preliminary understanding and represent an important step toward providing a foundation for more targeted programmes and future research initiatives aimed at older adults living in those areas. Geographically, the study is confined to only one municipality. Despite these limitations, we believe that the current approach was appropriate given the exploratory nature of the study. The information generated in this study serves as crucial input for the design of health policies and programmes aligned with national and regional priorities for older adults (37).

## 5 Conclusion

The study provides preliminary yet compelling evidence that the integration of satellite-based internet connectivity in teledentistry improves access to oral healthcare for geographically isolated populations. Combining a digital health platform with real-time remote consultations enables management of urgent and chronic oral health needs in difficult to access regions. The model's classification within the Industry 4.0 framework underscores its technological maturity and foundational role in Dentistry 4.0. Despite limited scope, its successful implementation suggests scalability and relevance. Future directions should include the integration of contextual artificial intelligence and expansion into other underserved areas, reinforcing the role of digital ecosystems in reducing health disparities.

## Data Availability

The raw data supporting the conclusions of this article will be made available by the authors, without undue reservation.
